# Hofmann vs. Paracelsus: Do Psychedelics Defy the Basics of Toxicology?—A Systematic Review of the Main Ergolamines, Simple Tryptamines, and Phenylethylamines

**DOI:** 10.3390/toxics11020148

**Published:** 2023-02-03

**Authors:** Luis Alberto Henríquez-Hernández, Jaime Rojas-Hernández, Domingo J. Quintana-Hernández, Lucas F. Borkel

**Affiliations:** 1 Unit of Toxicology, Clinical Science Department, Universidad de Las Palmas de Gran Canaria, Paseo Blas Cabrera Felipe s/n, CP 35016 Canary Islands, Spain; 2 Asociación Científica Psicodélica, CP 35300 Canary Islands, Spain; 3 Asociación Canaria para el Desarrollo de la Salud a través de la Atención, CP 35007 Canary Islands, Spain; 4 Faculty of Psychology, Universidad del Atlántico Medio, CP 35017 Canary Islands, Spain

**Keywords:** psychedelics, LSD, MDMA, mescaline, psilocybin, lethal dose, overdose, toxicology

## Abstract

Psychedelics are experiencing a strong renaissance and will soon be incorporated into clinical practice. However, there is uncertainty about how much harm they can cause at what doses. This review aimed to collect information on the health-hazardous doses of psychedelic substances, to be aware of the risks to which patients may be subjected. We focused on ergolamines, simple tryptamines, and phenylethylamines. We reviewed articles published in major medical and scientific databases. Studies reporting toxic or lethal doses in humans and animals were included. We followed PRISMA criteria for revisions. We identified 3032 manuscripts for inclusion. Of these, 33 were ultimately useful and gave relevant information about effects associated with high psychedelics doses. Despite having different molecular structures and different mechanisms of action, psychedelics are effective at very low doses, are not addictive, and are harmful at extremely high doses. For LSD and psilocybin, no dose has been established above which the lives of users are endangered. In contrast, MDMA appears to be the most dangerous substance, although reports are biased by recreational missuses. It seems that it is not only the dose that makes the poison. In the case of psychedelics, the set and setting make the poison.

## 1. Introduction

Today, psychedelics are back in the spotlight, at a very important time for public health due to the rising incidence of mental illness [1]. Governments now seem to accept the scientific evidence [2] and it appears that science will have the opportunity to resume research. However, much time has been wasted and research needs to be effectively focused. On the basis of the available information, it appears that psychedelic substances defy classic pharmacological models, calling into question even Paracelsus’ universal postulate that the dose makes the poison. Thus, basic research on psychedelics is needed, as well as translational and clinical research, which constitutes the main body of knowledge on these substances. In fact, little is known about the mechanism of action of many of these substances, as well as their metabolic pathways, all of which are necessary for the safe use of these potent chemicals.

**Figure 1:**
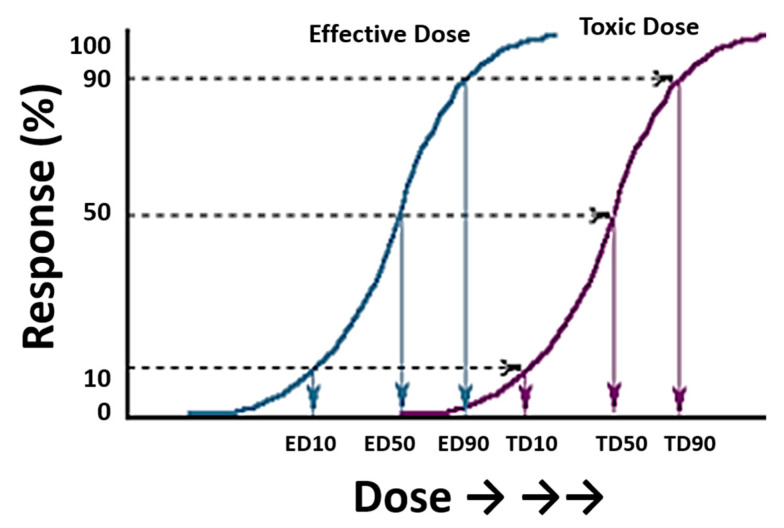
Typical sigmoid curves representing the dose of a drug and its response (measured as a percentage) in a group of individuals. The plot includes two curves relating to effective doses (left) and toxic doses (right). Extrapolation of the response gives the effective and toxic doses (ED and TD, respectively) for 10, 50, and 90% of the individuals exposed to these doses. Figure modified from the Toxicology Teaching Manual of the University of Las Palmas de Gran Canaria with the authors’ permission.

In general terms, the toxicity of a drug can be defined as the specific ratio between the active dose and the lethal dose [3]. This gives rise to various indices that can be calculated mathematically, with the lethal dose 50 (LD_50_) being the most important. LD_50_ is the amount of a substance that kills 50% of the individuals subjected to that substance, by a specific route of administration and for a defined species. The relationship between effectiveness and toxicity must be well studied in order to understand the risks and benefits of a medicine (Figure 1). In classical pharmacological and toxicological models, the effects observed in a population follow a normal distribution. Thus, 66% of the population will have an expected effect around the mean dose ± standard deviation (SD); 95% of the population will have an expected effect around the mean dose ± 2SD; and 99.8% of the population will have an expected effect around the mean dose ± 3SD, with sensitive individuals at the left extreme of the curve experiencing a high effect at low doses, and resistant individuals at the right extreme experiencing no effect even at high doses [4].

In the evaluation of dose response, pharmacogenetics—an area of pharmacology that is used to determine in advance what will be the best medicine or dose for an individual—is a key element. In that context, it is necessary to determine the main metabolization pathways of different substances, and it is important to determine how certain genetic variations affect the functioning and efficiency of these metabolization pathways (e.g., single-nucleotide polymorphisms (SNPs) or methylation status in cytochrome P450 family and other enzymes). Pharmacogenetics constitute an important tool for the interpretation of toxicological data, and can be crucial for determining the cause and modality of drug-related deaths [5], especially in cases of non-overdose of drugs of abuse [6]. Forensic pharmacogenetics is a field of toxicology not fully understood in general terms and almost unknown in the case of psychedelics.

Psychedelics had their golden age in the 1950s and the first half of the 1960s, when promising effects were observed in relation to the treatment of addictions and the resolution of psychological and psychiatric problems [7,8]. Despite this, their use was banned by the Richard Nixon administration in mid-1970s. These substances were stigmatized, giving rise to a period we have called the “Acid Panic”. During that period, many publications demonstrating the therapeutic power of psychedelic substances were censored, and even false or scientifically unsound articles were published [9]. As a consequence, society was imbued with a fear to psychedelics that has lasted for 50 years. Fortunately, scientific studies have continued, helping to dispel many of the myths surrounding these substances. Since the late 1980s, the use of psychedelics in the clinic has seen a renaissance, timid at first and very strong in recent years [10]. At present, there has been a translational evolution from the bench to the bedside, with phase 2 and 3 trials and/or evidence synthesis in particular. However, basic research (pharmacological and toxicological), which may not have been updated for decades, has taken a back seat. Thus, while the therapeutic potential of these substances—and their enormous potency—is well known, the reality is that the toxic/lethal doses of many of these substances are currently unknown [11]. Referring to mushrooms and LSD, “it’s virtually impossible to die from an overdose of them; they cause no physical harm; and if anything they are anti-addictive, as they cause a sudden tolerance which means that if you immediately take another dose it will probably have very little effect” [12,13]. Thus, do these substances defy the basic law of toxicology that says that the dose makes the poison?

The aim of the present review is to understand the potentially lethal doses of three groups of psychedelics (ergolamines, simple tryptamines, and phenylethylamines), in order to determine the safety of these substances and to understand their very special nature.

## 2. Materials and Methods

The search for articles was carried out in major medical and scientific databases from the early years of the 20th century to 31 August 2022, using the following search terms and Medical Subject Headings (MeSHs): psychedelic overdose, LSD overdose, MDMA overdose, LD_50_ LSD, LD_50_ MDMA, LD_50_ mescaline, LD_50_ psilocybin, psychedelic poisoning, death by LSD, death by MDMA, death by psychedelics, dosing psychedelics, and acute toxicity by psychedelics. The inclusion criteria were: (1) Original Article, (2) Case Report, (3) Review, (4) Articles in English, and (4) In vivo studies. Exclusion criteria were (1) In vitro studies, (2) abstract, (3) poster, and (4) communications at conferences. Two articles were written in a language other than English. In these cases, the abstract was translated to extract the most relevant information for this review. It should be noted that many of the clinical case reports were published in the 1960s and 1970s, when analytical methods were less developed. Articles that did not contain sufficient information to discern the role of psychedelic substances in the individual’s clinic were discarded.

**Figure 2:**
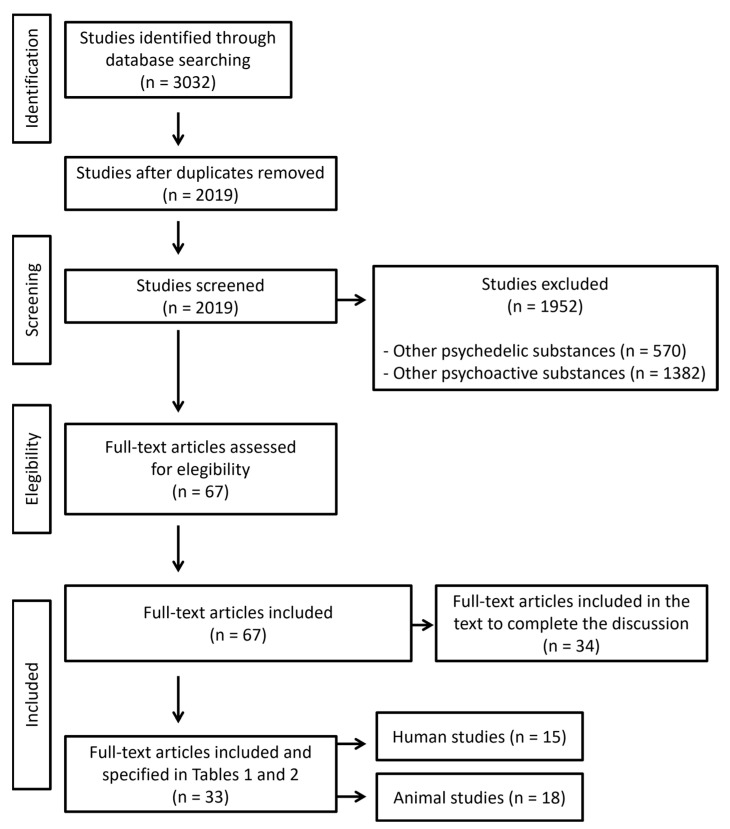
Flow diagram illustrating included and excluded studies in this systematic review. Articles reporting dosage are included in Tables 1 and 2 (n = 33), while articles reporting information that may explain clinical outcome are included in the text to complete the discussion (n = 34).

Once the search was conducted, studies reporting toxic or lethal doses related to psychedelics were included. We identified 3032 manuscripts for inclusion. The initial quality assessment was made evaluating the title and the abstract. We excluded articles referred to other psychedelic and psychoactive substances. After a screening and evaluation of the whole text, a total of 67 manuscripts were eligible: 33 gave relevant information about effects associated to high doses of psychedelics and 34 were included in the text to complete the discussion. Articles reporting experimental data on lethal doses in animals were also included. Characteristics of eligible studies are summarized in Figure 2. The revision was made according to PRISMA 2020 guidelines. The search was conducted between 1 July and 31 July 2022.

## 3. Results and Discussion

A total of 67 articles were included in this review. Of them, 33 gave relevant information about high/lethal doses of psychedelics. Eighteen studies (54.5%) were referred to high/lethal doses of psychedelics in animals and 15 (45.5%) were publications related to high/lethal doses of psychedelics in humans. Of them, 2 studies were experiments conducted directly on humans, 4 were reviews of clinical cases, and 9 were case reports. Additionally, 34 articles were included for being useful in the discussion of the main results.

### 3.1. Ergolamines: Does LSD Defy the Basics of Toxicology?

Lysergic acid diethylamide (LSD) is a semi-synthetic natural product created by Albert Hofmann at Sandoz Laboratories (Switzerland) in 1938. From the early days, studies were initiated to determine the toxicity of a product that was striking for its potency, achieving intense effects at very low doses. Despite being used by psychologists and psychiatrists, LSD was classified as an ‘experimental drug’, which prevented its use in clinical trials, in 1962 [14]. In 1965, its illegal production and sale were criminalized; in April 1966, Sandoz Laboratories stopped marketing LSD, and in 1968, possession and sale became a criminal offence [14]. In 1971, it was classified as a psychotropic drug under the Vienna Convention and banned. The period we have called “Acid Panic” began, during which an enormous effort was made to prove that LSD was harmful and highly toxic, mainly based on clinical cases reported in the scientific literature.

Table 1 summarizes experiments related to high/lethal doses of psychedelics in animals. The elephant was found to be the most sensitive animal, as administration of 0.06 mg/kg caused the death of one of these animals in 1962 [15]. However, the experiment was repeated years later, in 1984, without any consequence [16]. The LD_50_ in mice was 50–60 mg/kg; 16.5 mg/kg in rats; 0.3 mg/kg in rabbits [9,17,18]. In all cases, LSD was administered intramuscular or intravenously. Although there are important inter-species differences, LSD appeared to be a very potent substance when administered in this way, which is not the route of administration for humans. LSD has been tested in other species (i.e., Guinea pig or wild birds), at different doses and by different routes of administration, but no reliable conclusion has been reached as to a dose above which it is lethal [19,20]. Given that the effective dose in humans is 0.001–0.003 mg/kg, it can be inferred that the LD_50_ for our species could be 300–600 times that of the rabbit and up to 50,000–100,000 times that of the mouse [9]. It was deduced that LSD was remarkably well tolerated by humans, on whom it nevertheless exerted intense effects at very low doses.

Table 2 summarizes clinical reports related to high/lethal doses of psychedelics in humans. In 1973, the first article on LSD overdose was published [21]. It concerned eight patients (four men and four women), aged between 19 and 39 years. Of these, four were tested for LSD in their blood, showing concentrations between 0.0021 and 0.026 μg/mL. Two individuals—not tested for blood—had LSD in their gastric contents, and reported having snorted the substance. Half of the patients were also positive for ethanol and/or cocaine. This heterogeneity makes it difficult to determine what role LSD may have played in the individual’s medical history. In addition, it is necessary to relate the concentration detected in blood to the dose of LSD taken.

The first pharmacokinetic study of LSD dates back to 1972. After oral administration of 160 μg of LSD, the blood concentration of the substance was observed to be 4.16 ng/mL two hours later [22]. Similar results were obtained later [23,24], allowing an approximation of the maximum amount taken of more than 1.5 mg. For the eight reported patients in 1972, this is 7 times the maximum effective dose (250 μg [25]), which would presuppose moderate-severe intoxication. However, all patients survived with no adverse health consequences.

Four years later, a case of death from LSD overdose was reported in which 31.2 μg/mL of LSD was quantified in the liver of the deceased. The authors inferred, on the basis of cat studies, that the deceased must have received an extrapolated dose of 320 mg intravenously (1600 times the recommended dose) [26]. This particular death is one of two probable cases of death by LSD overdose [17]. The other case was reported in 1985 as follows. Under the title “A fatal poisoning with LSD”, the authors reported the death of a 25-year-old male who died, allegedly, from the action of the substance [27]. Analyses were performed by radioimmunoassay, a semi-quantitative technique with limited expert value, giving a concentration of 14.8 and 4.8 ng/mL before and after death. High-performance liquid chromatography analysis, considered the gold standard, gave a result of 8 ng/mL before the individual’s death. According to pharmacokinetic studies, this may result in an exposure to about 500 μg of LSD, slightly more than twice the maximum recommended effective oral dose. Although, for some substances, doubling or tripling the effective dose may pose an undisputed health risk (i.e., hypoglycemics, cytostatics, or anticoagulants), for LSD, numerous cases of massive overdose without health consequences have been reported [11]. One of the most extreme cases is that of a 46-year-old woman who snorted 55,000 μg of LSD—275 to 550 times the maximum recommended effective dose—mistaking it for cocaine. The incident had no adverse health consequences. Moreover, she overcame an opiate addiction shortly afterward [28].

The literature is full of studies reporting LSD intoxications, with hundreds of individuals included [29,30,31] and no serious cases with fatal outcome reported. However, the message given during the “Acid Panic” era was that LSD was extremely dangerous and should remain banned. Since then, the scientific community has published cases and studies in the opposite direction: LSD is not only a safe molecule, due to its wide margin of safety, but also showed no addiction potential [32]. In 1993, the LD_50_ was set as 14,000 μg [32], which was then raised to 100,000 μg in 2004 [17], which is 400 times the maximum dose commonly used in the therapeutic setting, without empirical evidence. Even taking this theoretical value for granted, it is difficult to think of drugs or medicines that, when administered 100 or 200 times their therapeutic dose, do not cause serious damage to the health of the individual.

While it is true that there have been reported cases of deaths where LSD was present, they are all related to violent incidents—police intervention and aggressive restraint measures—where the victims had made missuses of the substance, not because of the substance, but because of the experience: inappropriate places and inappropriate circumstances [11]. The scientific community now recognizes that LSD is an extremely safe substance when used in moderate doses (50–250 μg) in controlled settings and orally, with only modest elevations in blood pressure, heart rate, and body temperature [33,34].

Dosing brings up an all-important question that applies to any substance under scrutiny: pharmacologically speaking, what is the drug’s toxicity? Hofmann himself knew that the substance was effective at very low doses. Substances that are effective at low doses usually have very narrow safety margins. Interestingly, in the case of LSD, not only are there no adverse health effects, reported incidents at very high doses have had no serious or irreversible consequences, and these facts call into question Paracelsus’ toxicological principle that the dose makes the poison.

### 3.2. Phenylethylamines

#### 3.2.1. Does MDMA Defy the Basics of Toxicology?

3,4-methylenedioxy-methamphetamine (MDMA) was synthesized by the E. Merck pharmaceutical firm in Darmstadt (Germany) in 1912. The patent was registered on 24 December of that year and came into force on 16 May 1914 (number 274350) [35]. It was included as an experimental drug by the US government in its ‘truth serum’ mind control program [36] and was the subject of research by psychiatrists, led by Alexander Shulgin [37]. However, although clinical trials showed promising results [38,39], MDMA was banned by the Federal Drug Administration (FDA) in July 1985.

MDMA has a number of particularities that mean it needs to be treated specifically. First, it has non-linear kinetics, which means that there is no linear correspondence between the dose ingested, the amount of MDMA in blood, and the physiological effects [40]. Second, the metabolites generated in the metabolization of the drug cause an inhibition of CYP2D6, which is the main MDMA-metabolizing enzyme, exposing the individual to drug intoxication and overdose of MDMA itself, in the case of repeated ingestions [40]. The effective oral dose in humans is 1–2 mg/kg [17,25]. Even at moderate doses, there are a number of potential health risks including cerebral hyperthermia, hyponatremia, or disseminated intravascular coagulation. However, the wide variety of adverse effects it can produce leads experts to believe that there must be factors other than the substance itself that explain these effects: environmental conditions in places where the substance is taken recreationally, the quality of the synthesis in home laboratories, or impurities added to maximize the benefits [41]. In addition, the metabolization of MDMA is carried out mainly by CYP2D6, whose genetic polymorphisms condition the efficiency of metabolization. Although the role of these polymorphisms is not entirely clear, they may have an important influence on a fatal outcome, although it is likely that several factors are required concomitantly [42].

MDMA can be injected, smoked, or snorted, but is usually ingested orally. The LD_50_ of MDMA via intraperitoneal administration has been reported as 97, 49, and 98 mg/kg for the mouse, rat, and guinea pig, respectively (Table 1). The LD_50_ of MDMA via intravenous administration has been reported as 22, and 14 mg/kg for the monkey and dog, respectively [43]. The LD_50_ of MDMA via oral administration has been reported with a range from 160 mg/kg [44] to 325 mg/kg [45] among rats.

There are many case reviews of MDMA-overdose-related deaths in the scientific literature [46], although no clear conclusions can be drawn: (i) in most publications, it was not possible to definitively know the role of the substance in the death; (ii) the concentrations of MDMA in the deceased were unknown; (iii) when available, it is difficult to infer the dose taken because the time between intake and death was unknown [42]. In a series of 392 cases reported in Australia between 2000 and 2018, an average of 0.45 mg/L of MDMA was detected [47]. In a series of 142 cases reported in Norway between 2000 and 2019, an average of 0.73 mg/L of MDMA was detected, although 36% of the cases had other drugs besides MDMA [48]. The lethal concentration found in 27 MDMA-related deaths was 3 mg/L, reporting a high influence of environmental factors [17]. A reasonable estimate of the acute LD_50_ of MDMA for a healthy 70 kg person would appear to be approximately 2 g, or about 15–16 times a single recreational oral dose of 125 mg/kg [49].

**Table 2:** Description of clinical reports related to high/lethal doses of psychedelics in humans.

Substance	Year	N	Gender	Type of publication	Dose/[Blood]	Route	Outcome	LD/LD_50_	Author
LSD	1943	1	M	Sandoz Laboratory	0.25 mg	oral	Survive		Hofmann
	1974	7	M/F	Recreational use	0.026 µg/mL	in	Survive		Klock et al.
	1977	1	NA	Case report	31.2 µg/mL *	oral	Death	320 (mg)	Griggs et al.
	1985	1	M	Case report	0.008 µg/mL	NA	Death	0.6 (mg)	Fysh et al.
	1993	NA	M/F	Clinical case reviews		oral	Survive	14 (mg)	Gable
	2020	1	F	Recreational use	0.5 mg	oral	Survive		Haden et al.
		1	F	Recreational use	1.2 mg	oral	Survive		
		1	F	Recreational use	55 mg	in	Survive		
MDMA	2004	27	M/F	Clinical case reviews	3 mg/L	oral			Gable
	2020	392	M/F	Clinical case reviews	0.45 mg/L	oral			Roxburg et al.
	2022	142	M/F	Clinical case reviews	0.37–0.73 mg/L	oral			Jamt et al.
Mescaline	1962	10	M	Experiment	2.5 mg/kg	im	Survive		Wolbach et al.
	1985	1	NA	Case report		NA	Death	9.7 (mg/L)	Reynolds et al.
	1993	NA	M/F	Clinical case reviews		oral	Survive	6000 (mg)	Gable
	1999	1	M	Case report		oral	Death	0.48 (mg/L)	Nolte et al.
Psilocybin	1960	16	NA	Experiment	60 µg/kg	oral	Survive		Hollister et al.
		16	NA	Experiment	37 µg/kg	ip	Survive		
	1962	10	M	Experiment	75 µg/kg	im	Survive		Wolbach et. al
	1993	NA	M/F	Clinical case reviews		oral	Survive	14,000 (mg)	Gable
	1996	1	M	Case report	6000 mg	oral	Death	4 (mg/L)	Gerault et al.
	2012	1	F	Case report		oral	Death	30 (µg/L)	Lim et al.

Abbreviations: M, male; F, female; in, intranasal; im, intramuscular; ip, intraperitoneal; NA, not available. * Found in liver.

MDMA is a drug with low addictive power but with certain risks, essentially linked to the way it is taken [32]. Despite its massive and uncontrolled use, a fatal incident risk of 0.003% (1 in 33,000 pills) has been estimated, lower than other illegal drugs of abuse consumed in the same context [50], which makes it a very safe drug. To maximize its therapeutic benefits, it is not only the dosage that must be appropriate, but also the circumstances and setting of the treatment. In this scenario, no significant adverse effects or deaths have been reported in individuals who have taken the substance in the context of guided therapy. MDMA is, however, a strange case for two reasons. First, despite being more harmful to health than other psychedelics, it is, along with ketamine, among the substances that are closest to being approved for clinical use. It has to be taken into account that MDMA, together with ketamine and ibogaine, are distinguished from classic psychedelics, both in their effects and in their pharmacology [51]. Secondly, although there is a large literature on MDMA-related deaths, the lethal dose orally administered under controlled conditions is unknown.

#### 3.2.2. Does Mescaline Defy the Basics of Toxicology?

Natural psychedelics such as mescaline or psilocybin are found in plants considered sacred since ancient times. In the case of mescaline (trimethoxyphenethylamine), it is found in San Pedro (*Echinopsis pachanoi*) and Peyote (*Lophophora williamsii*) cacti. Although they have been used by indigenous tribes for centuries, they reappeared in Europe and the United States in the mid-1950s [52]. In any case, mescaline was already being investigated by the American government years before its immersion in the society of that time [36].

Mescaline is the least toxic of the methoxyamphetamines tested in animal models [53], and is 2500–4000 times less potent than LSD [54]. The toxic effects of trimethoxyphenethylamine were investigated for the first time in 1934, reporting an LDL_0_ (Lethal Dose Low) of 500 and 750 mg/kg in the Guinea pig and frog, respectively [55]. In the 1960s, several studies were published in mice, reporting an LD_50_ between 157 and 880 mg/kg depending on the route of administration [56,57,58,59] (Table 1). While for the rat, the reported LD_50_ was 15 mg/kg, among monkeys, the dose reached 130 mg/kg, which is 8.6 times more [43,60]. The LD_50_ via the oral route—the main route of administration in humans—in animals reaches almost 1 g/kg [17]. In any case, none of these values can be extrapolated to the human species, mainly because the animal studies use trimethoxyphenethylamine and the intake in humans is a preparation of the cactus, which implies the intake of many other substances with the interactions inherent to it.

The first study in humans began in 1921 and ran for several years. Sixty subjects (90% males) were injected with 200, 400, 500, and 600 mg of pure mescaline. The results were published in an extensive document entitled Der Meskalinrausch (Mescaline intoxication) by Kurt Beringer in 1927 [61]. Physical and psychological reactions were described as “mescal psychosis”, with no relevant adverse effects or deaths reported. The TDL_0_ was set at 2.5 mg/kg (intramuscular administration), according to Wolbach in 1962. The experiment was made in 10 males who were morphine addicts serving sentences for violations of the U.S. national narcotic laws. The authors concluded that reactions induced by LSD, mescaline, and psilocybin were qualitatively similar, with no relevant adverse effects or deaths reported [62].

Two case reports were published in 1985 and 1999 with fatal outcome. The first one was a subject who fell from a cliff while they were under the influence of mescaline. Concentrations of the drug were 9.7, 70.8, and 1163 µg/mL or µg/g in the blood, liver, and urine, respectively [63]. Concentrations in the blood and the actions described by eyewitnesses presuppose that the deceased was suffering from the hallucinogenic effects of mescaline, whose hallucinogenic effects are acquired at doses of 200–500 mg of the salt [63]. The second one was a 32-year-old Native American man with a history of alcoholism who died from bronchial aspiration of vomit during a Peyote ceremony [64]. Antemortem blood concentration was 0.48 mg/L (Table 2). Mescaline has potent emetic effects [65]; therefore, it should be used with caution if there are esophageal or respiratory pathologies or concomitant use of central nervous system depressants (i.e., alcohol). To our knowledge, no further cases of mescaline-associated death have been reported.

From these two cases, it cannot be inferred a dose from which the consumer’s life is in danger, especially if the consumption is performed in a controlled environment [66]. The usual effective dose (and range) for non-medical purposes is 350 mg (200–450) [17]. Although mescaline is assigned a lower safety ratio than other classical psychedelics [17], it has pharmacokinetic properties that make it especially safe. The plasma half-life of mescaline is approximately 6 h [67]. Its low lipid solubility means that it crosses the blood–brain barrier more slowly, is stored temporarily in the liver, and is released slowly, reducing its potential adverse effects [67]. Thus, the peak of psychological effects, which occurs 2 h after ingestion, does not coincide with the peak of mescaline concentration in the brain. In general terms, mescaline is very poorly absorbed orally. It is estimated that 60% of the substance consumed is eliminated unchanged in urine one hour after ingestion [68]. Unlike other psychedelics, the mechanism of action of mescaline is not known. Hallucinogenic effects are believed to be due to stimulation of serotonin and dopamine receptors in the central nervous system.

If we add to all this the way it is taken—preparations and cooking of the cactus—where many other alkaloids interact, mescaline should be taken with caution. Even so, it is a substance that has been used for centuries and that, even today, is an elemental part of the culture of some tribes. However, no serious cases with fatal endings have been reported [54,69], which again brings into question the basic principle of toxicology.

### 3.3. Tryptamines: Does Psilocybin Defy the Basics of Toxicology?

Natural psychedelics such as psilocybin ([3-[2-(dimethylamino)ethyl]-1H-indol-4-yl] dihydrogen phosphate) is found in so-called magic mushrooms (Psilocybe semilanceata, P. cubensis or Pholiotina cuanopus). It is a substance that has been used since ancient times and was reintroduced into Western culture in the mid-1950s after the publication, in *Life Magazine*, of R.G. Wasson’s psychedelic experiences with magic mushrooms, in Mexico, guided by María Sabina in 1957 [70].

Some of the most potent psychedelics belong to the group of tryptamines (i.e., N,N-dimethyltryptamine (DMT) or N,N-dimethyl-5-methoxytryptamine (5-MeO-DMT)) and, curiously, they are among those with the greatest margin of safety [17]. Regarding psilocybin, studies in animal models show a high tolerance up to 400 mg/kg, with the substance proving highly toxic at higher concentrations [71]. In 1968, the LD_50_ in mice was established at 275 and 420 mg/kg intravenously and intraperitoneally, respectively [59]. Similar results were reported later [71]. The rabbit was shown to be the most sensitive species, in which the LD_50_ intravenously was 13 mg/kg, while for the rat, it was 280 [72] (Table 1).

For the human species, 30–40 mg is considered to be a high dose of psilocybin [25], which, in comparison with the data reported in animals, suggests that we are a particularly sensitive species. the TDL_0_ was established at 60 and 37 µg/kg orally and intraperitoneally, respectively, in a group of 16 volunteer subjects tested in 1960 [73]. By the intramuscular route, the TDL_0_ was 75 µg/kg (Table 2), highlighting that reactions induced by LSD, mescaline, psilocin, and psilocybin are qualitatively similar [62].

Two case reports were published in 1996 and 2012 with fatal outcome. The first one report a case that occurred in France in 1993 [74]. A quantity of 4 ng/mL of psilocybin was found in the blood. However, the work appears to be invalidated by numerous methodological deficiencies and contradictions [75], and was highly controversial and criticized by some sectors of French society. The second one is a fatal case of magic mushroom ingestion in a heart transplant recipient, who collapsed 2–3 h after the intake [76]. Plasma toxicology revealed a psilocin level of 30 mg/L and a tetrahydrocannabinol level of 4 mg/L. No alcohol or other common drugs of abuse were detected. The cause of death was determined to be psilocin toxicity. The toxicity of psilocybin is low (LD_50_ = 280 mg/kg in rats); a 60 kg person would need to ingest up to 17 kg of fresh mushrooms to reach this dose. However, psilocybin toxicity includes cardiovascular toxicity; therefore, beyond the dosage, it must be taken into account that the individuals have a good health and body condition before ingestion. There is a third publication reporting a death associated to psilocybin, but details are scanty [77]. There are other deaths reported as a result of accidents or self-harm following mushroom ingestion [78], which are beyond the scope of this review.

In the United States, approximately over one million people have used mushrooms without fatalities [79]. Similar outcomes have been reported in Europe [80], which makes magic mushrooms a safe substance at different doses, from micro-doses to so-called heroic doses [78]. Under controlled circumstances, psilocybin has a wide margin of safety. Although the dose makes the poison, in order for these potent transformative substances to be dangerous to humans, Paracelsus’ own principle is called into question.

### 3.4. Limitations of the Study

Although this systematic review was made according to PRISMA 2020 guidelines, some limitations were present. First, many studies published in non-indexed journals were not included; second, many studies published in non-selected databases were not included; third, no statistical analysis was performed; fourth, the biological reasons behind the particular behavior of these substances can only be hypothesized.

With respect to the latter, is important to highlight the role of pharmacogenetics studies in drug-related overdoses and deaths [5], especially in the case of MDMA, the most risky psychedelic among those included in this review, whose complex metabolism is linked to CYP2D6. Thus, determining the presence of cytochrome inducing or suppressing mutations can provide answers in cases of death related to a suspected drug overdose. This is especially relevant if we take into account that these substances are not only used in assisted therapies but, increasingly, for personal growth mainly due to its neuroenhancement effects [81]. Although there are conflicting opinions regarding their actual functioning and benefit [82], it seems that psychedelics report benefits even in microdoses [83]. In any case, the effects that these practices may have when receiving higher doses of psychedelics in relation to tolerance, metabolization efficiency, and other parameters related to the pharmacokinetics of the substances are not known.

## 4. Conclusions

Despite their therapeutic potential in psychology and psychiatry, psychedelics have been subjected to severe scrutiny that led to their prohibition in the 1960s and 1970s. They are currently undergoing what is called a ‘psychedelic renaissance’, being the subject of extensive research—especially clinical research—and are close to legalization in many parts of the world (e.g., the State of Oregon, USA). Despite their high potency—they are capable of very potent actions at very low doses—they have very low addiction rates and very high safety rates, especially the classic psychedelics (LSD, mescaline, or psilocybes). In some cases, a 100-fold increase in the effective dose does not cause harmful effects on the health of individuals, which defies the basic principle of toxicology. Perhaps with the exception of MDMA, historically reported deaths do not appear to be the sole responsibility of the substance, but rather related to the environment and circumstances of intake. In order to understand this unique behavior, pharmacogenetics may be crucial. In view of the current situation regarding psychedelics, there is a need to invest in basic research, which clarifies the pharmacokinetics of these substances as well as their mechanism of action.

In view of the results shown in this review, it seems that it is not only the dose that makes the poison; in the case of psychedelics, the set and setting also make the poison.

## Figures and Tables

**Table 1:** Description of experiments related to high/lethal doses of psychedelics in animals.

Substance	Year	Species	Dose/[Blood]	Route	LD/LD_50_ (mg/kg)	Author
LSD	1957	Rabbit	0.3 mg/kg	iv		Rothlin
	1959	Rat	17 mg/kg	iv		Gable **
	1959	Mouse	46 mg/kg	iv	100 *	Gable **
	1962	Elephant	297 mg	iv	14 *	West et al.
	1962	Guinea pig	16 mg/kg	sc		De Jonge
	1972	Bird	1.8 mg/kg	oral		Schafer
	1984	Elephant	0.003–0.10 mg/kg	oral		Siegel
MDMA	1973	Mouse		ip	97	Hardman et al.
		Rat		ip	49	
		Guinea pig		ip	98	
		Dog		iv	14	
		Monkey		iv	22	
	1985	Rat		oral	325	Goad
	1997	Rat	20–360 mg/kg	oral	160	De Souza
Mescaline	1934	Guinea pig		sc	500	Grace
		Frog		p	750	
	1961	Mouse (50)		oral	880	Greenblatt et al.
	1962	Mouse		sc	534	Hoshikawa
	1968	Mouse		iv	157	Horibe
	1968	Mouse		NA	261	Walters et al.
	1973	Mice (40)		ip	212	Hardman et al.
		Rat (28)		ip	13	
		Guinea pig (32)		ip	328	
		Dog (16)		iv	54	
		Monkey (17)		iv	130	
	1985	Rat		sc	534	Becker
		Rat		iv	15	
		Rat		im	330	
	2004	Mouse		oral	880	Gable
Psilocybin	1968	Mouse		ip	420	Horibe
		Mouse		iv	275	
	1972	Rabbit		iv	13	Usdin
		Rat		iv	280	
	2015	Mouse	200–450 mg/kg	ip	316.9 Ⴕ	Zhuk et al.

Abbreviations: M, male; F, female; iv, intravenous; sc, subcutaneous; ip, intraperitoneal; p, parenteral; im, intramuscular; NA, not available. * Estimated value from the experiment (mg). ** Details of the original manuscript were obtained from Gable, 2004. Ⴕ For *Psilocybe semilanceata*. LD_50_ for *Psilocybe cyanopus* was 316.9 mg/kg. LD_50_ for psilocin was 293.1 mg/kg.

## Data Availability

Not applicable.
